# Multiphoton Absorption Simulation of Sapphire Substrate under the Action of Femtosecond Laser for Larger Density of Pattern-Related Process Windows

**DOI:** 10.3390/mi12121571

**Published:** 2021-12-17

**Authors:** Xintian Cai, Chaoyue Ji, Changkai Li, Zhiqiang Tian, Xuan Wang, Cheng Lei, Sheng Liu

**Affiliations:** 1 The Institute of Technological Sciences, Wuhan University, Wuhan 430072, China; caixintian@whu.edu.cn (X.C.); 2018106520021@whu.edu.cn (C.J.); tianzhiqiang@whu.edu.cn (Z.T.); 2 School of Physics, Peking University, Beijing 100871, China; lichangkai@pku.edu.cn; 3 Beijing National Laboratory for Condensed Matter Physics, Institute of Physics, Chinese Academy of Sciences, Beijing 100190, China; xw@iphy.ac.cn; 4 School of Power and Mechanical Engineering, Wuhan University, Wuhan 430072, China

**Keywords:** femtosecond laser, sapphire, multiphoton absorption, transient nonlinearity

## Abstract

It is essential to develop pattern-related process windows on substrate surface for reducing the dislocation density of wide bandgap semiconductor film growth. For extremely high instantaneous intensity and excellent photon absorption rate, femtosecond lasers are currently being increasingly adopted. However, the mechanism of the femtosecond laser developing pattern-related process windows on the substrate remains to be further revealed. In this paper, a model is established based on the Fokker–Planck equation and the two-temperature model (TTM) equation to simulate the ablation of a sapphire substrate under the action of a femtosecond laser. The transient nonlinear evolutions such as free electron density, absorption coefficient, and electron–lattice temperature are obtained. This paper focuses on simulating the multiphoton absorption of sapphire under femtosecond lasers of different wavelengths. The results show that within the range of 400 to 1030 nm, when the wavelength is large, the number of multiphoton required for ionization is larger, and wider and shallower ablation pits can be obtained. When the wavelength is smaller, the number of multiphoton is smaller, narrower and deeper ablation pits can be obtained. Under the simulation conditions presented in this paper, the minimum ablation pit depth can reach 0.11 μm and the minimum radius can reach 0.6 μm. In the range of 400 to 1030 nm, selecting a laser with a shorter wavelength can achieve pattern-related process windows with a smaller diameter, which is beneficial to increase the density of pattern-related process windows on the substrate surface. The simulation is consistent with existing theories and experimental results, and further reveals the transient nonlinear mechanism of the femtosecond laser developing the pattern-related process windows on the sapphire substrate.

## 1. Introduction

The development of microwave and optoelectronic devices has led to stricter requirements for semiconductor materials. The third-generation wide-bandgap semiconductors represented by GaN, SiC, and diamond can meet the requirements of harsh environments, and therefore, they have received increased attention [1,2,3]. However, the dislocation density of wide-bandgap semiconductors can be as high as 10^8^–10^10^ cm^−2^ with a significant impact on electronic mobility and other properties, which seriously hinders their application and development [4,5]. Therefore, it is particularly important to reduce the dislocation density to improve the growth quality of the wide bandgap semiconductor film. Taking GaN as an example, numerous studies have shown that sapphire is one of the suitable substrates for film growing, and lateral epitaxy is the most effective method to reduce its dislocation density. When utilizing lateral epitaxial growth, it is necessary to develop pattern-related process windows on a sapphire substrate. Most of the dislocations will be truncated and disappear after a 90° lateral bending [6,7,8,9].

In lateral epitaxial growth technology, researchers generally use mask photolithography or chemical etching to process the surface structure on the sapphire substrate. Due to the short pulse time, extremely high peak fluence, and high absorptivity compared with the nanosecond and picosecond laser, the femtosecond laser has been increasingly applied in the processing of substrate patterns.

However, the transient nonlinear process of materials under the action of the femtosecond laser is complex, which is affected by the wavelength, pulse width, spot size, intensity, and other laser parameters. In addition, it is closely related to the material’s dielectric constant, photon absorption coefficient, reflectivity, lattice-specific heat, and other characteristics. Existing studies have shown that the free electron density on the crystal surface is the principal physical feature of the early transient evolution, which has a non-negligible effect on the processes [10,11]. In addition, the threshold concentration of free electrons for ablation simulations can obtain consistent results with traditional thermal ablation [12,13,14]. Stuart et al. studied the laser processes with different parameters acting on insulators and proposed a free electron theory [15]. For the processes of ultrashort pulses acting on the insulator, Shirk et al. conducted a more systematic study: (1) A two-step heat transfer model was proposed and simulated by molecular dynamics; (2) taking the laser intensity and absorption rate into consideration, the electronic heat distribution was predicted within a picosecond; (3) the ablation threshold electron temperature and equilibrium temperature was predicted; (4) the ablation volume and size of the heat-affected zone in the material were predicted [16]. L. Jiang et al. studied the ablation depth of femtosecond lasers acting on molybdenum disulfide, which also improved the two-temperature model (TTM) that simulates the evolution of electron–lattice temperature [17,18]. W. Hu, H.R. Wang, N.M. Bulgakova established a laser–plasma nonlinear transient coupling model. The transient process of free electron density under the action of the femtosecond laser was studied to reveal the influence of the femtosecond laser on the optical properties of the diamond surface [19,20,21]. Maolu Wang et al. simulated and analyzed the ultrafast process of sapphire crystals under the action of the femtosecond laser based on the free electron density theory and conducted experimental verification [22,23]. Existing studies have shown that changes in laser parameters will change the material’s physical properties, such as the multiphoton absorption coefficient, avalanche ionization coefficient, and ablation threshold concentration [24,25]. However, there is still a lack of research on the multiphoton absorption process of sapphire crystals under the action of femtosecond lasers of different wavelengths. This involves the dynamic evolution of free electrons, photon–electron–lattice energy transfer, and transient nonlinear processes of ablation on the picosecond to femtosecond scale. Moreover, there are still difficulties in experimentally revealing the underlying mechanism adequately. In particular, the transient nonlinear processes of multiphoton absorption at different wavelengths need to be further revealed.

Based on the above research, the Fokker–Planck equation was adopted to simulate the process of femtosecond laser-induced generation of free electrons. Simultaneously, the TTM equation was used to simulate the heat transfer process between the electrons and the lattice. The distributions of the free electron density, absorption coefficient, electron, and lattice temperature were obtained. The ablation process of sapphire material under the action of the femtosecond laser was studied. In addition, the transient nonlinear process of multiphoton absorption under different femtosecond laser wavelengths was simulated, which provides a theoretical basis for developing higher density pattern-related process windows for improving the growth quality of wide-bandgap semiconductor films.

## 2. Methods

### 2.1. The Fokker–Planck Equation for Solving Free Electron Density

The free electron density inside the crystal is not only related to the initial incident laser, such as intensity, pulse width, spot size, peak power, peak power reaching time, and other parameters, but also related to the reflectivity and absorption coefficient of the material [22,26].

Because in the range of investigated wavelengths the sapphire crystal is almost transparent and has a wide bandgap, the energy of a single photon is inadequate to excite free electrons. While the peak power of femtosecond lasers is extremely high, the number of photons incident on the material instantaneously is significantly large. The electrons in the crystal can absorb the energy of multiple photons and jump up to the conduction band. When absorbing the photon energy, the electrons are excited to a higher energy level on the same conduction band. The electrons—at higher energy levels—will return to the bottom of the conduction band through in-band transition, which stimulates the electrons in the valence band to jump up to the conduction band. This forms an avalanche reaction, which causes the electron concentration to rise exponentially. The avalanche ionization in the conduction band occurs on the order of femtoseconds. When the laser pulse width is greater than 50 fs, the avalanche ionization cannot be ignored [27,28]. According to the Fokker–Planck equation, the free electron density of the crystal can be given as:

(1) $$\frac{\partial n_{e}}{\partial t}=\alpha_{i}I+\delta_{m}I^{m}-n_{e}/\tau$$

where $n_{e}$ is the free electron concentration; t is time; $\alpha_{i}$ is the avalanche ionization coefficient; I is the femtosecond laser intensity; $\delta_{m}$ is the multiphoton absorption coefficient; m is the multiphoton number; τ is the electron decay time.

In Equation (1), avalanche ionization, multiphoton absorption, and free electron decay are mainly considered. The multiphoton absorption coefficient is mainly determined by the laser frequency, the number of absorbed photons required for ionization, and the multiphoton absorption cross-section coefficient. Avalanche ionization is mainly determined by the material’s avalanche ionization cross-section coefficient and refractive index. The inelastic collision loss is related to the material properties. The electron decay time of sapphire is 150 ps, which does not vary with laser parameters [22].

### 2.2. The TTM Equation for Solving the Electron and Lattice Temperature

Since the width of the laser is on the order of femtoseconds and the electroacoustic coupling time is on the order of picoseconds, the laser energy is first transferred to the electrons. The electrons heat up and energy is transmitted to the lattice through electroacoustic coupling. The electron–lattice two-temperature model is used to represent the heat transfer process [17,29].

(2) $$c_{e}\frac{\partial T_{e}}{\partial t}=\nabla \left(\kappa_{e}\nabla T_{e}\right)-g\left(T_{e}-T_{1}\right)+S$$

(3) $$c_{1}\frac{\partial T_{1}}{\partial t}=\nabla \left(\kappa_{1}\nabla T_{1}\right)+g\left(T_{e}-T_{1}\right)$$

where $c_{e}$ is the electron specific heat; $c_{1}$ is the lattice specific heat; $T_{e}$ is the electron temperature; $T_{1}$ is the lattice temperature; $\kappa_{e}$ is the electronic thermal conductivity; $\kappa_{1}$ is the lattice thermal conductivity; g is the electron lattice coupling coefficient; S is the laser thermal fluence. The laser thermal fluence has been defined as the energy source through electron heating and determined by $S=\frac{\alpha_{i}\cdot I}{n_{e}}$. The TTM equation couples the temperatures of the electron and the lattice. Temperature and laser energy are linked by laser thermal density S. Combined with the Fokker–Planck equation in Section 2.1, the laser thermal density is mainly affected by the free electron density.

### 2.3. Ablation Judgment for Nonthermal Melting

As the free electron density increases, the absorption coefficient of the laser inside the crystal also increases. When the free electron density reaches the ablation threshold, the laser energy is rapidly deposited. The chemical bonds between atoms are broken and the crystal lattice is irreversibly damaged. Then, nonthermal ablation occurs. The threshold concentration of free electrons is given by the following formula [30,31]:

(4) $$N_{cr}=\frac{\omega^{2}*\varepsilon *m_{e}}{e^{2}}$$

where ω is the frequency of femtosecond laser; ε is the dielectric constant; $m_{e}$ is the electron mass; e is the electronic charge.

The existing theory has proved that the results of using the threshold concentration of free electrons as the ablation standard and the traditional melting temperature as the standard are similar [31].

## 3. Simulation

**Figure 1:**
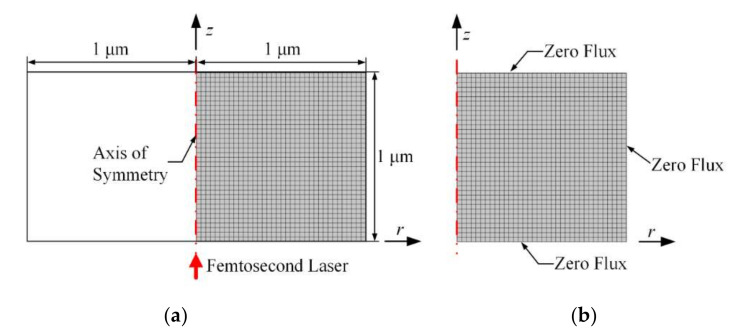
Simulation model. (**a**) Grid Division; (**b**) boundary condition.

Based on the above theory, we have established a simulation model of the multiphoton absorption of the femtosecond laser acting on sapphire. Since the femtosecond laser is a three dimensional (3D) axisymmetric Gaussian beam, it is considered on the two dimensional (2D) r-z plane for simplicity. The surface structure of the substrate material is generally on the order of nanometers. Therefore, the simulation area is set to be in a cylinder, as shown in Figure 1a. Considering the accuracy and efficiency of the calculation, the mesh type is set as a free quadrilateral mesh controlled by the user, and the calibration is a refined semiconductor. The analysis domain is refined into a rectangular mesh to discretize, and the temperature field is calculated at the unit node. Except for the symmetry axis without setting boundary conditions, the remaining three edges are set to zero flux boundary conditions, as shown in Figure 1b. The ambient temperature is 300 K. The femtosecond laser has a pulse width of 100 fs, wavelength of 800 nm, and fluence of 4 J/cm^2^. The time step is 1 fs, and the total calculation time is 10 ps.

The finite element method is used to perform numerical calculations on the model. First of all, the main parameters in our simulation are determined according to the mechanism of femtosecond laser and sapphire, as shown in Table 1. Afterward, the coupling equations are solved to obtain the free electron density, laser power, reflectivity, absorptivity, electron temperature, lattice temperature, and other physical quantities in the r-z 2D plane. Moreover, the ablation morphology is judged by the threshold concentration of free electrons.

**Figure 2:**
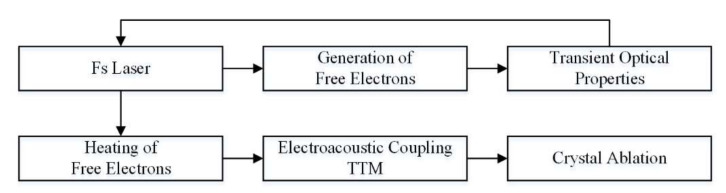
Energy coupling of femtosecond laser acting on sapphire.

In the equations, the electron temperature and lattice temperature are described by the two-temperature model. The two-temperature model was originally used to reveal the femtosecond-scale thermal transfer mechanism when the femtosecond laser acts on metal. As the mechanism of femtosecond laser action has been continuously and deeply studied, the two-temperature model has been extended to the process of the femtosecond laser acting on insulators and semiconductors. Compared with metals, when the femtosecond laser acts on sapphire, the laser energy will not only heat electrons but also generate free electrons, which mainly involves multiphoton absorption and avalanche ionization. The energy coupling of the femtosecond laser acting on sapphire is shown in Figure 2.

Our simulation process comprises the following steps: (1) calculate the free electron density, the absorption coefficient, and reflectivity according to the Fokker–Planck equation; (2) calculate the temperatures of the electrons and the lattice according to the TTM equation; (3) determine the ablation morphology according to the threshold concentration of free electrons; (4) adjust the laser wavelength, calculate the number of multiphoton needed for ionization, and correct the absorption coefficient for further simulation.

## 4. Results and Discussion

**Figure 3:**
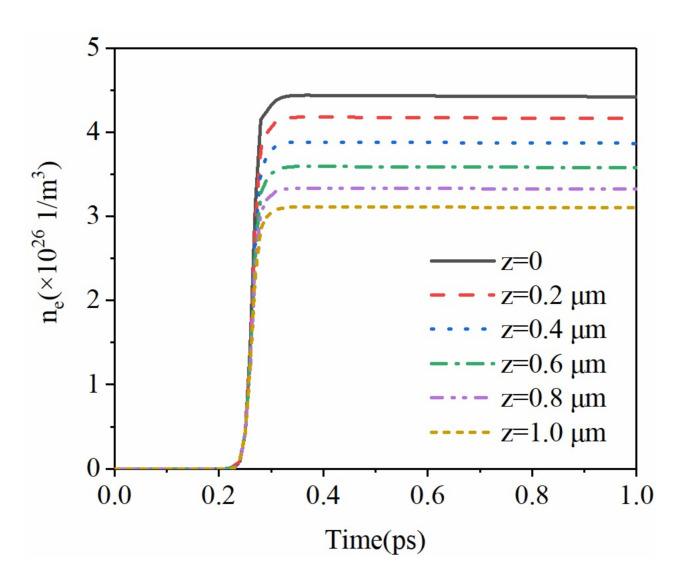
Variation of free electron density with time at different depths at *r* = 0.

**Figure 4:**
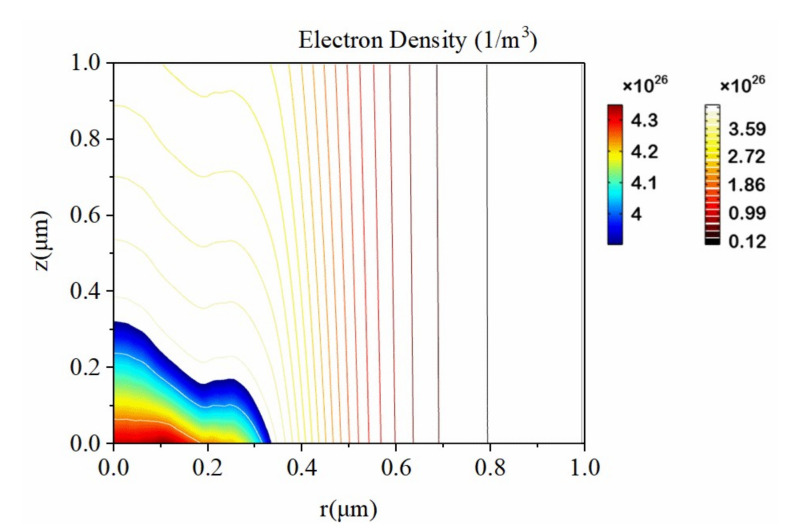
Spatial distribution of free electron density at *t* = 300 fs.

The change in electrical and optical properties of materials under the action of the femtosecond laser is caused by the absorption of photon energy. When the femtosecond laser acts on the crystal surface, since the electroacoustic coupling time is usually on the order of picoseconds, the photon energy is first transferred to the electrons. The nonfree electrons inside the crystal ionize after absorbing the energy of the photons. With a 4 J/cm^2^ laser intensity, the evolution of the free electron density at different depths in the sapphire is shown in Figure 3. Before 200 fs, the free electron density is extremely low. After 200 fs, the free electron density rises sharply with a continual occurrence of multiphoton absorption and avalanche ionization. The free electron density on the outermost surface (*z* = 0) reaches the threshold first. As the depth increases, the free electron density gradually decreases. When the free electron density exceeds the threshold, the material is determined to be ablated. The spatial distribution of free electron density at 300 fs is shown in Figure 4. We observe that the farther away from the laser center, the lower the free electron density, and the distribution is roughly Gaussian. At 300 fs, the free electron density at *r* = 0, *z* = 0 reaches $4.3\times 10^{26}\;m^{-3}$, which is highly consistent with the results in Figure 3.

**Figure 5:**
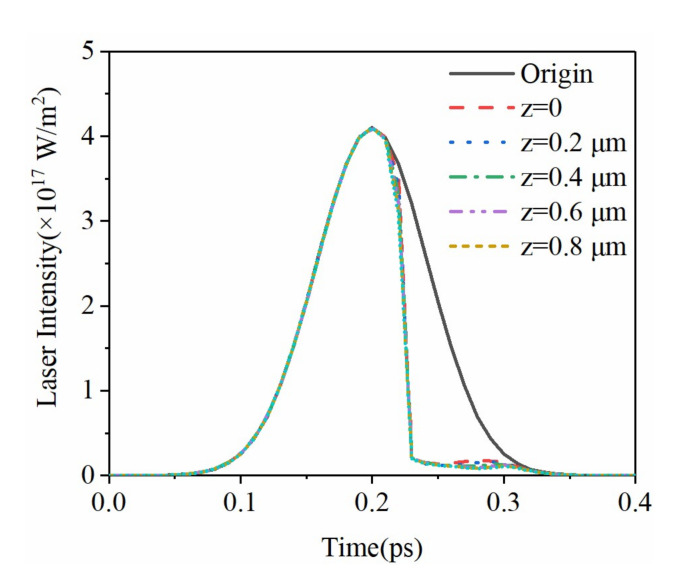
Distribution of femtosecond laser intensity in time domain at *r* = 0.

**Figure 6:**
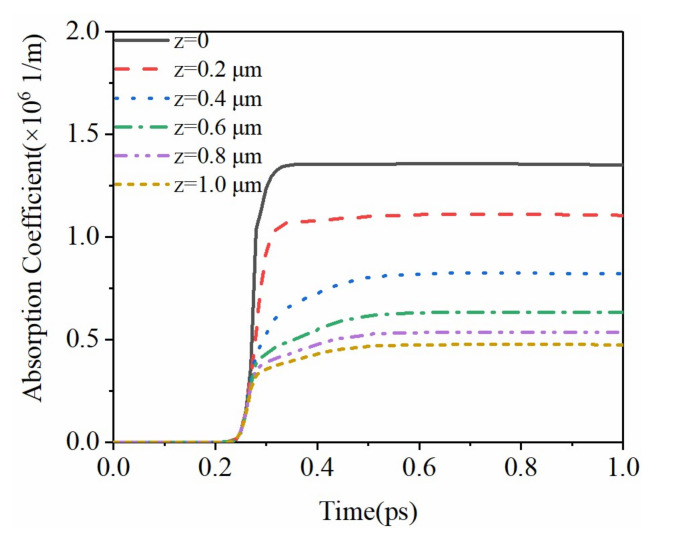
Variation of absorption coefficient.

The intensity of the initial incident laser is related to the pulse width, spot size, pulse loading time, and peak power. The laser intensity inside the material not only depends on the parameters of the incident laser but is also related to the absorption coefficient and the reflectivity. The evolution of the laser intensity is shown in Figure 5. Combined with the evolution of the free electron density in Figure 3, the free electron density generated by multiphoton absorption is extremely low before 200 fs. The pulse duration here refers to the full width at half maximum (FWTH). For Gaussian distribution, FWTH is approximately 2.355 times the standard deviation. At this time, the reflectance and absorption coefficients of the material are very small, and the crystal can be regarded as transparent. Therefore, the influence of laser intensity by depth can be ignored, and it is consistent with the initial incident laser. The laser power reaches the peak at 200 fs. Then, as the free electron density increases sharply, the optical properties gradually approach as metal [32]. While the reflectivity increases rapidly, the laser intensity on the surface and inside of the sapphire drops to nearly zero. Figure 6 shows the evolution of the absorption coefficient inside the material. Combined with the analysis in Figure 3, the absorption coefficient is closely related to the rate of change of the free electron density. At 240 fs, the absorption coefficient at different depths inside the crystal drops to nearly zero. The corresponding free electron density reaches the maximum and lasts for picoseconds. This is because of the long decay time of free electrons in sapphire crystals, which is negligible in the femtosecond time scale.

**Figure 7:**
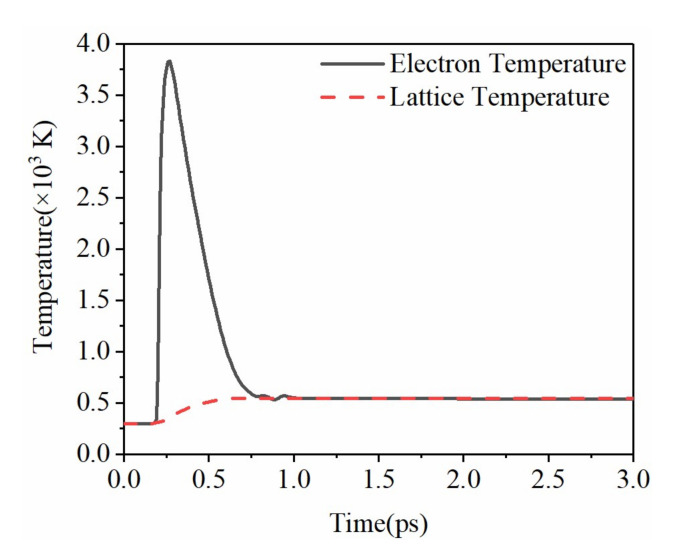
Temperature of electron and lattice.

Since the pulse width of the femtosecond laser is smaller than the electroacoustic coupling time, the laser energy is first absorbed by electrons, then transferred to the crystal lattice by energy coupling between the electron and the lattice [31]. Figure 7 shows the evolution of electrons and lattice temperature. Analyzing the evolution of free electron density in Figure 3, in the initial stage, due to the lower free electron density, the rising temperature of the electron and lattice is not obvious. After 200 fs, due to the substantial occurrence of avalanche ionization, the free electron density rises sharply, and the temperature of the electrons rises to as high as 3800 K. At this moment, the change in the lattice temperature is relatively slow since that the electroacoustic coupling time is usually on the order of picoseconds. This tremendous difference creates the temperatures of the electron and lattice in two different temperature systems in the initial picoseconds. With the end of the laser action and the generation of electroacoustic coupling, the electron temperature is continuously reduced to be consistent with the lattice temperature. 

**Figure 8:**
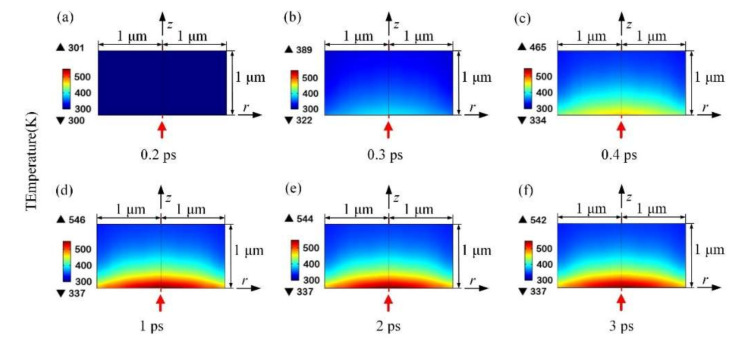
Lattice temperature at different times. (**a**) 0.2 ps (**b**) 0.3 ps (**c**) 0.4 ps (**d**) 1 ps (**e**) 2 ps (**f**) 3 ps.

Figure 8 shows the lattice temperature at different times in space. We observe that the lattice temperature always presents a Gaussian distribution in space, which is due to the energy of the femtosecond laser being Gaussian in time and space. Moreover the heat conduction between the lattices—due to the temperature difference within picoseconds—can be neglected.

The electron heating is in the femtosecond time scale, and the lattice heating is in the picosecond time scale, while the heat conduction in the lattice is generally in the nanosecond time scale. As shown in Figure 7, when the femtosecond laser is acting on the sapphire crystal, the free electron density on the surface of the material reaches the ablation threshold at 300 fs and the electron temperature could be as high as 3800 K. At this moment, the energy has not yet been transferred to the lattice. However, the high-density and high-energy free electrons break the chemical bonds in the lattice due to the Coulomb force, and the crystal surface explodes into particle plasma. Distinct from the thermal ablation, the Coulomb explosion already occurs when the heat is transferred to the lattice. The ultrafast process occurs on a sub-picosecond time scale and does not involve electroacoustic coupling and lattice heat conduction, so it is called a “nonthermal” process.

However, the mechanism of femtosecond laser ablation is very complicated, and not only involves the nonthermal process but also the thermal process. The thermal process includes nonthermal equilibrium vaporization and melting, while the nonthermal process includes Coulomb explosion and electrostatic ablation. These processes either coexist or vary according to changes in processing conditions. Currently, researchers have not reached a consensus on the mechanism. In this paper, the threshold concentration of free electrons theory is used to determine the ablation of sapphire.

**Figure 9:**
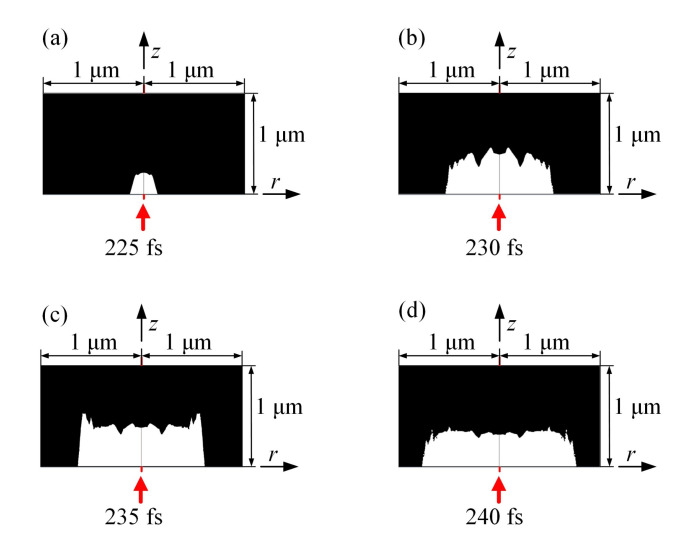
Morphology evolution of ablation pit based on threshold concentration of free electrons. (**a**) 225 fs femtosecond laser exposure (**b**) 230 fs femtosecond laser exposure (**c**) 235 fs femtosecond laser exposure (**d**) 240 fs femtosecond laser exposure

When the free electron density increases to the ablation threshold, pits will appear on the crystal surface [33]. According to Formula (4), the threshold concentration of free electrons of sapphire is $3.9\times 10^{26}\;m^{-3}$ under the 800 nm wavelength femtosecond laser. According to the threshold concentration of free electrons theory, the morphology of ablation pits can be calculated, as shown in Figure 9. At 225 fs, the ablation has just occurred, and the ablation area is concentrated near the light spot and roughly has a Gaussian distribution. At 230 fs, the ablation area is obviously larger and the bottom is close to Gaussian distribution. At 235 fs, the bottom of the ablation pit is relatively flat and no longer presents a Gaussian distribution. At 240 fs, the bottom of the ablation pit is flatter and the ablation pit does not increase significantly. The final ablation pit has a depth of 0.34 μm and a radius of 0.8 μm.

**Figure 10:**
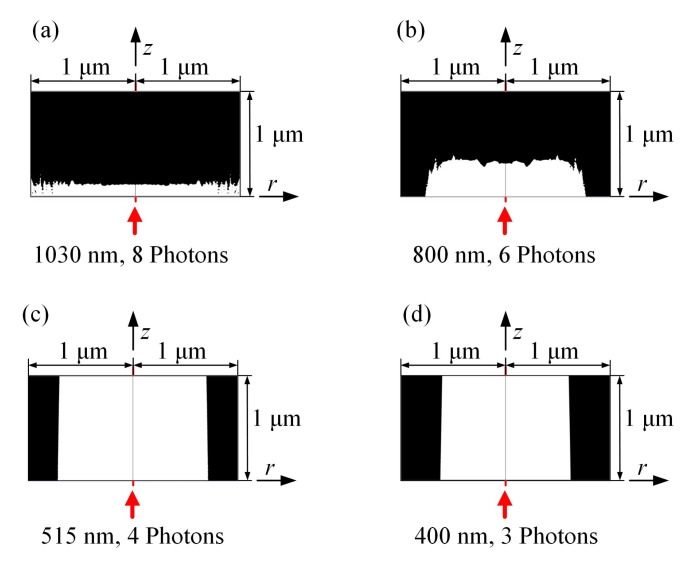
Ablation pit shape based on threshold concentration of free electrons under different number of absorbed photons. (**a**) 1030 nm laser wavelength, 8 photons required (**b**) 800 nm laser wavelength, 6 photons required (**c**) 515 nm laser wavelength, 4 photons required (**d**) 400 nm laser wavelength, 3 photons required.

In order to further explore the influence of femtosecond laser parameters on the ablation pits of sapphire, we chose femtosecond lasers with four wavelengths: 1030, 800, 515, and 400 nm for simulation, corresponding to eight, six, five, and four-photon absorption. Table 2 lists the diameter and depth of the sapphire pits formed by multiphoton absorption at four wavelengths. The depth of the ablation pit during eight-photon absorption was 0.10 μm and the diameter was 1 μm. The depth of the ablation pit during six-photon absorption was 0.34 μm and the diameter was 0.8 μm. The depth of the ablation pit during four-photon absorption was greater than 1 μm and the diameter was 0.7 μm. The depth of the ablation pit during three-photon absorption was greater than 1 μm and the diameter was 0.6 μm. The morphology of the ablation pit is shown in Figure 10. Inside the material, black means not ablated, and white means ablated. The results show that the larger the laser wavelength, the corresponding increase in the number of photons required for ionization, the larger the radius of the ablation pit obtained, and the smaller the depth. This trend is more obvious at wavelengths above 500 nm. When the wavelength is below 500 nm, the ablation is deeper. This is mainly because as the laser wavelength decreases, the number of multiphoton required for ionization decreases and the ablation becomes easier.

In our simulation, using a 1030 nm laser wavelength, 100 fs pulse width and 4 J/cm^2^ laser power, the ablation depth was 0.10 μm and its radius was 1 μm. In existing theories and experiments, when the other conditions are the same, with laser wavelength of 1030 nm, pulse width of 255 fs and laser power of 6 J/cm^2^, the ablation depth is 0.14 μm and its radius is 1 μm [22]. As shown in Table 3, the 0.04 μm difference in ablation depth was mainly caused by different laser power and pulse width [22]. This result is highly consistent with existing theories and experiments. Therefore, our results of multiphoton absorption at different wavelengths show good accuracy and credibility.

## 5. Conclusions

By solving the Fokker–Planck partial differential equation and the TTM gradient coupling equation, the distribution or evolution of physical quantities such as the free electron density, absorption coefficient, and electron–lattice temperature in sapphire is obtained. The ablation of sapphire under the action of femtosecond laser was studied. We emphatically simulated the transient nonlinear process of multiphoton absorption of sapphire under the action of femtosecond lasers of different wavelengths. The results show that within the action time of hundreds of femtoseconds, the free electron density rose to $4.2\times 10^{26}\;m^{-3}$, the laser intensity rose to $4\times 10^{7}\;W\cdot m^{-2}$ and then dropped to nearly zero, and the absorption coefficient increased to $1.3\times 10^{6}\;m^{-1}$. Within several picoseconds, the electron temperature first rose to 3800 K and then dropped to 500 K, while the lattice temperature rose from 300 K to be the same as the electron temperature. Within the range from 400 to 1030 nm, the larger the wavelength, the larger the number of multiphoton required for ionization, and wider and shallower ablation pits could be obtained. The smaller the wavelength, the smaller the number of multiphoton, and narrower and deeper ablation pits could be obtained. This is consistent with existing theoretical and experimental results. Under the simulation conditions presented in this paper, the minimum ablation pit depth can reach 0.11 μm, and the minimum radius can reach 0.6 μm.

The simulation further reveals the transient nonlinear processes of the electron–lattice when the femtosecond laser acts on the sapphire substrate. In the range of 400 to 1030 nm, selecting a laser with a shorter wavelength can obtain pattern-related process windows with a smaller diameter, which is beneficial for increasing the density of pattern windows on the substrate surface, and has theoretical guiding significance for reducing the dislocation density of GaN film growth.

## Figures and Tables

**Table 1:** Main parameters and initial values.

Main Parameters	Initial Values
Decay time constant of electron τ	150 ps
Required photon number for multiphoton ionization m	6
$\mathrm{Femtosecond}\;\mathrm{laser}\;\mathrm{intensity}\;I_{0}$	$4\times 10^{13}\;W/{\mathrm{cm}}^{2}$
$\mathrm{Sapphire}\;\mathrm{band}\;\mathrm{gap}\;E_{g}$	8.8 eV
Sapphire refraction index n	1.75
$\mathrm{Electron}\;\mathrm{collision}\;\mathrm{time}\;\tau_{c}$	4 fs
Avalanche ionization cross-section coefficient σ	$2.39\times 10^{17}\;{\mathrm{cm}}^{2}$

**Table 2:** Sapphire ablation under multiphoton absorption.

**Femtosecond Laser Wavelength (** nm **)**	**Multiphoton Number**	**Ablation Depth (** μm **)**	**Ablation Radius (** μm **)**
1030	8	0.10	1.0
800	6	0.34	0.8
515	4	>1	0.7
400	3	>1	0.6

**Table 3:** Comparison of simulation and experimental results Data from [22].

Laser Parameters	Wavelength (nm)	Pulse Width (fs)	Power (J/cm^2^)	Ablation Depth (μm)	Ablation Radius (μm)
Simulation	1030	100	6	0.14	1.0
Experiment	1030	255	4	0.10	1.0

## Data Availability

Not applicable.
